# Advancements and prospects for eco-friendly, high-performance silver bismuth halide solar cells†

**DOI:** 10.1039/d4sc07955h

**Published:** 2025-03-06

**Authors:** Natalia Belen Correa Guerrero, M. Dolores Perez, Naoyuki Shibayama, Tsutomu Miyasaka

**Affiliations:** a Toin University of Yokohama 1614 Kurogane-cho, Aoba Yokohama Kanagawa Japan shibayama@toin.ac.jp miyasaka@toin.ac.jp; b Depto. Física Materia Condensada (GIyA), Instituto de Nanociencia y Nanotecnología (CONICET), CNEA, Centro Atómico Constituyentes Avda. Gral. Paz 1499, San Martín 1650 Buenos Aires Argentina mdperez@unsam.edu.ar; c Research Center for Advanced Science and Technology (RCAST), The University of Tokyo 4-6-1 Komaba, Meguro-ku Tokyo Japan

## Abstract

The demand for lead-free alternatives to lead-halide perovskite (LHP) solar cells has prompted extensive research efforts to explore alternative materials. Silver bismuth iodide (Ag–Bi–I) absorbers have an appropriate band gap between 1.8 and 1.9 eV for solar cells and exhibit a high absorption coefficient and excellent stability under ambient conditions. However, achieving sufficient power conversion efficiency (PCE) at the lab scale. The maximum PCE reported to date for Ag–Bi–I (SBI) materials is 5.56%, a much lower PCE value than those obtained for LHP based solar cells. Various approaches have been employed to improve the properties of SBI-based solar cells, including solution engineering, additive incorporation, and cation exchange. However, trap-assisted recombination and intrinsic limitations may be the underlying factors impacting their efficiency. With an overview of previous research efforts on SBI materials, we highlight different approaches for PCE enhancement and discuss the current state of basic research on material preparation and analysis. Furthermore, this study offers insights and prospects for SBI as a material for solar energy applications.

## Abstract 2

We introduce the current challenges, improvement strategies, and future prospects of lead-free solar cells using silver bismuth halide, which is expected to be a promising candidate for high-efficiency lead-free solar cells.

## Graphical abstract

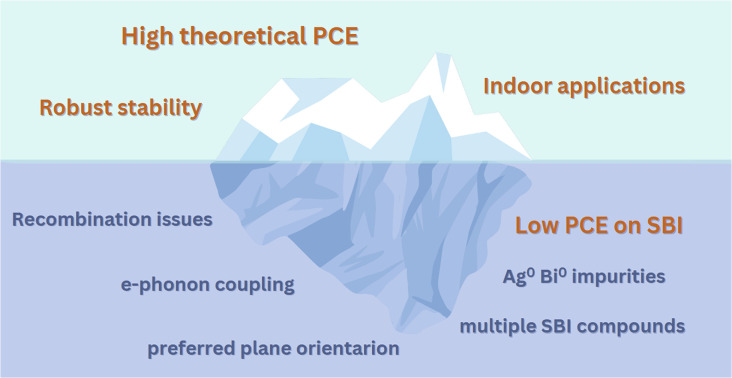

## Introduction

Lead halide perovskite (LHP) solar cells have demonstrated remarkable progress over the past 15 years since the publication of our group's seminal research paper,^1^ achieving a power conversion efficiency (PCE) exceeding 26%.^2^ Notably, silicon/perovskite tandem solar cells have achieved a PCE of 34%.^3^ This significant advancement positions perovskite solar cells as a leading candidate for renewable energy adoption. Despite their significant potential for photovoltaic applications, several issues still need to be resolved before large-scale production becomes a reality.^4^ One major concern is their poor stability,^4,5^ as LHPs are known to be sensitive to environmental conditions. Strategies such as the development of low-dimensional perovskites and the application of passivation layers are employed to prevent degradation, thereby extending their durability.^6,7^ This is necessary not only for high performance, but also to reduce environmental impact by preventing external leakage of lead. Given the significant environmental impact associated with lead leakage, extensive research has been undertaken focusing on sealing technologies to mitigate such leakage, recycling strategies,^8^ and the development of lead-free perovskite solar cells.^9,10^ Double perovskites,^11^ cesium-based perovskite solar cells,^12,13^ and tin-based perovskite solar cells^14,15^ are some of the possible lead-free alternatives to LHP solar cells. While LHPs exhibit a promising trajectory, the quest for lead-free alternatives to LHPs is gaining momentum.

**Fig. 1:**
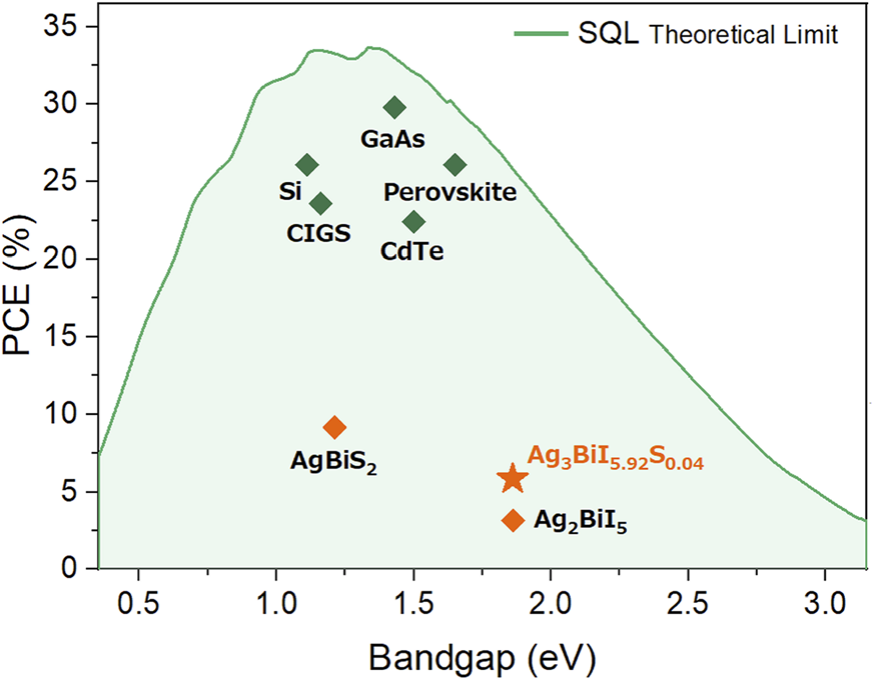
Power conversion efficiency *vs.* band gap energy, illustrating the highest efficiencies for various solar cells.

Following the pioneering work of Kim *et al.* in 2016 ^16^ and the subsequent demonstration by Turkevych *et al.* in 2017 ^17^ of the feasibility of using silver bismuth iodide (SBI) in solar cells, interest in employing lead-free materials with structural compositions distinct from those of perovskites has significantly increased for solar cell applications.^17^ The composition of SBI, inspired by LHP, is Ag_*a*_Bi_*b*_I_*a*+3*b*_, and these crystal structures exhibit Rüdorffite structures. Owing to their inorganic nature, these materials demonstrate superior moisture resistance compared to LHP solar cells. By modulating the silver iodide (AgI) and bismuth iodide (BiI_3_) ratio, SBI compounds can be easily tailored, ranging from Ag-rich to Bi-rich, including Ag_3_BiI_6_, Ag_2_BiI_5_, AgBiI_4_, and Ag_2_BiI_7_. As a result, SBI provides the capability to adjust the band gap from 1.8 eV to 1.9 eV and to modify conductivity from p-type (Ag-rich) to n-type (Bi-rich) states.^18^ Furthermore, both cations Bi^3+^ and Pb^2+^ have an ns^2^ electronic configuration and exhibit a high absorption coefficient. For instance, Ag_2_BiI_5_ exhibits a band gap of 1.86 eV,^19^ and the theoretical efficiency of a solar cell based on this material, as determined by the Shockley–Queisser limit (SQL), is approximately 25% with a short-circuit current density (*J*_sc_) of 17.99 mA cm^−2^ and an open-circuit voltage (*V*_oc_) of 1.56 V. At first glance, SBI appears to be a material with a promising future as a solar cell material. However, the highest conversion efficiency achieved in SBI-based solar cells is merely 5.56%^20^ and the average values of *J*_sc_ and *V*_oc_ range between 6.25 and 2.86 mA cm^−2^ and 0.66 and 0.54 V, respectively. According to the calculated SQL as shown in Fig. 1, the low experimental PCEs suggest large thermal losses for both *J*_sc_ and *V*_oc_ of approximately 75% and 47%, respectively. Various strategies, including solution engineering, additive incorporation, and cation exchange, have been explored to reduce these losses.^21–24^ Trap-assisted recombination has been observed in the presence of bismuth, leading to investigations aimed at reducing the recombination to improve performance by substituting Bi^3+^ with Cu^2+^ or Sb^3+^ cations.^25,26^

Despite extensive the efforts to enhance efficiency, the persistent inability to achieve superior outcomes, such losses might indicate an intrinsic issue with SBI that limits performance despite optimization attempts, necessitating a comprehensive understanding. However, before investing more time and effort into improving SBI, it is important to ask: is it necessary? What should be our next step in this process?

Despite the growing interest in SBI-based solar cells, research on these materials has often been overshadowed by broader studies on various lead-free alternatives. Many reports provide only a brief overview of Ag–Bi–I compounds, focusing on their potential and describing their properties, rather than addressing the fundamental challenges that limit their performance.^27–29^ As a result, critical issues specific to silver bismuth halides, particularly SBI, remain insufficiently explored.

In this work, we present a comprehensive review of research efforts dedicated to Ag–Bi–I materials, highlighting the strategies employed to enhance their photovoltaic performance. We also examine the current state of fundamental research in this area, integrating the latest findings. Additionally, we provide our perspective on the future of these materials to offer a broader view of the challenges and opportunities in the field.

## State of the art

It is accepted that SBI solar cells operate on a principle similar to that of lead-based perovskite solar cells, closely aligning with the configurations of n–i–p and p–i–n solar cells. In these designs, SBI functions as the intrinsic absorber (i), sandwiched between two selective contacts (p- and n-type). In the n–i–p structure, materials such as TiO_2_ (either compact or mesoporous) and SnO_2_ ^19,30,31^ serve as the n-type electron transport layer (ETL), while the hole transport layer (HTL) can be composed of polymeric materials such as poly[bis (4-phenyl)(2,4,6-trimethylphenyl)] amine (PTAA),^21,32^ 2,2′,7,7′-tetrakis[*N*,*N*-di(4-methoxyphenyl)amino]-9,9′-spirobifluorene (spiro-OMeTAD),^33,34^ poly(3-hexylthiophene-2,5-diyl) (P3HT),^35–37^ or inorganic materials like NiO,^38^ serving as the p-type contact. Starting with a transparent substrate such as indium tin oxide (ITO) or fluorine doped tin oxide (FTO), the complete structure typically follows the sequence: FTO or ITO/ETL/SBI/HTL/metal contact. Furthermore, in a p–i–n type structure, also known as an inverted structure, the intrinsic absorber is sandwiched between a p-type material (*e.g.*, NiO_*x*_) at the bottom and an n-type layer (*e.g.*, the combination of [6,6]-phenyl-C_61_-butyric acid methyl ester (PCBM)/C_60_) at the top, typically arranged as FTO or ITO/NiO_*x*_/SBI/PCBM/C_60_/metal contact. There have been reports of studies utilizing carbon electrodes *in lieu* of the C_60_/PCBM combination.^23,39^

SBI crystals are unique as they can be achieved using any stoichiometric ratio of Ag : Bi : I. Their fabrication has been explored through various techniques such as spin-coating, blade coating,^32^ printable ink coating,^23^ and solid-state reaction.^40^ Among these, the spin-coating method is the most commonly employed method for fabricating SBI crystalline layers. The first report of SBI layer preparation used butylamine as the solvent.^16^ Subsequently, mixed solvents of dimethyl sulfoxide (DMSO) and *N*,*N*-dimethylformamide (DMF) have been utilized in the preparation of SBI layers,^41^ and this solvent combination has become the primary choice. However, the optimal DMSO : DMF ratio for fabricating SBI layers remains controversial.^25,31,36,42^ Shadabroo *et al.* reported that the BiI_3_–DMSO complex intercalates with AgI molecules during the formation of SBI, thereby promoting crystallization.^34^ They also found that a DMSO : DMF ratio of 1 : 1 is the most suitable for obtaining the best performance.^34^ However, different ratios, such as 1 : 3, 3 : 1, and 3 : 2, are also effective for synthesizing SBI. ^19,20,25^ The use of hydriodic acid (HI)^22,37,40^ and methanol^33^ has been reported to improve the crystallinity of the SBI layer. Furthermore, employing these methods to increase the Bi to I ratio in SBI has been documented.^19,42^ The antisolvent method has also proven to be effective for obtaining high-quality SBI polycrystalline films, with the use of chlorobenzene,^36,43^ isopropanol (IPA),^30^ and toluene.^30^ This widely used method for the preparation of high-quality lead halide perovskite polycrystalline films^44,45^ can also enhance morphology and crystallinity in SBI layers. Airflow has also been studied to control solvent removal during this process.^20^ All the aforementioned methods are solution-based and often lead to the appearance of AgI as an impurity in the SBI film.^17–22^ Jung *et al.* reported the production of Ag_2_BiI_5_ as a stable phase under vacuum and high temperatures, avoiding the presence of AgI.^40^ This finding suggests that the low solubility of AgI in organic solvents leads to its precipitation during the crystal growth process of the SBI layer.^40^

**Fig. 2:**
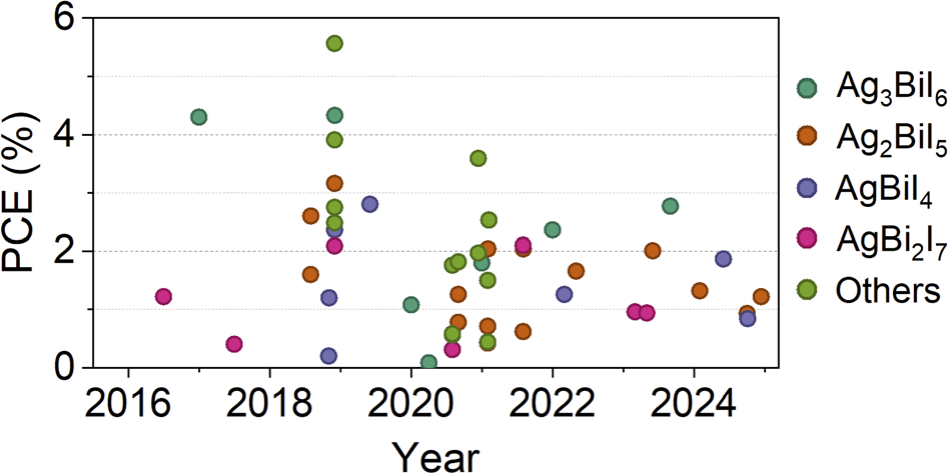
Efficiency of Ag_3_BiI_6_, Ag_2_BiI_5_, AgBiI_4_, Ag_2_BiI_7_, and other SBI-related compounds reported over the years (full table is given in ESI, Table S1†).

**Fig. 3:**
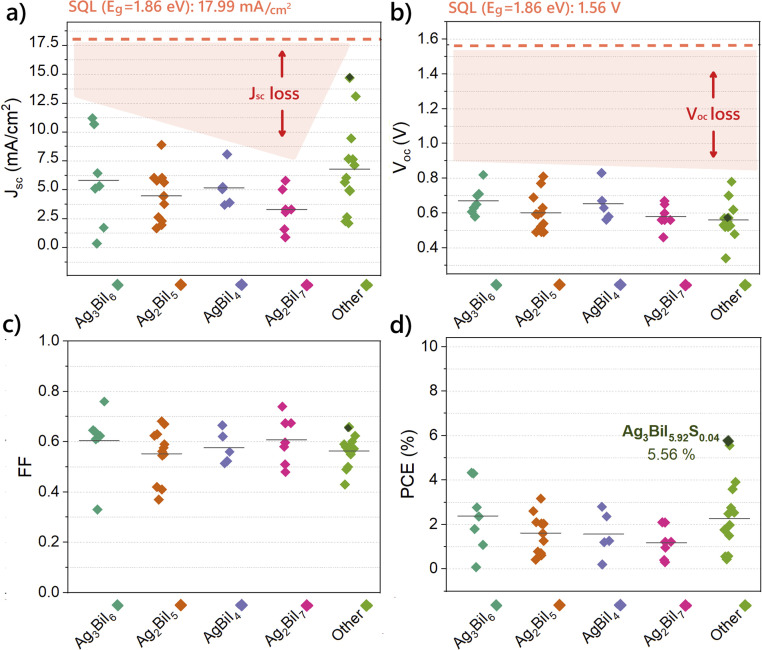
Electrical parameters *J*_sc_ (a), *V*_oc_ (b), FF (c), and PCE (d) for the most common SBI compounds Ag_3_BiI_6_, Ag_2_BiI_5_, AgBiI_4_, Ag_2_BiI_7_, and other SBI-related compounds reported over the years (full table is given in ESI, Table S1†). For reference, dashed lines refer to the values of the SQL theoretical limit for Ag_2_BiI_5_ (*E*_g_: 1.86 eV^19^). Parameters of the champion SBI solar cell are marked in dark green.

It is observed that various device configurations and solvent engineering strategies have been explored to optimize SBI-based solar cells. To provide a clearer perspective on these efforts and their impact on device performance, Fig. 2 presents the evolution of PCE over the years for different types of SBI compounds. Notably, the category labelled “Others” includes materials with mixed structures, SBI-based absorbers with partial substitution, or compositions deviating from the most well-studied compounds. As illustrated in Fig. 2, despite the diverse approaches employed, there remains significant room for improvement in efficiency, with the average PCE remaining around 2%. To assess the progress of SBI absorbers thus far, we have compiled the electrical parameters (*J*_sc_, *V*_oc_, fill factor (FF), and PCE) for the most commonly utilized SBI materials: Ag_3_BiI_6_, AgBiI_4_, Ag_2_BiI_5_ and AgBi_2_I_7_. This compilation provides insights into the various strategies adopted that have led to significant improvements (see Fig. 3 and Table S1†). For reference, we have marked the theoretical limits of an absorber with a band gap of 1.86 eV, corresponding to Ag_2_BiI_5_.^19^

One notable observation from Fig. 3 is that among all parameters, the *J*_sc_ exhibits the greatest deviation, yet it has the potential to approach the SQL.^20,51^ For all compounds, the *V*_oc_ generally varies between 0.5 eV and 0.8 eV, while the FF ranges from 0.5 to 0.7. These factors contribute to a low PCE, which averages at 1.75%. However, recent advancements have enabled SBI solar cells to achieve values up to 2%.^19,25,32,42^

It is known that recombination at the interface can detrimentally affect *V*_oc_. Previous researchers have shown that the addition of a spike layer is effective to reduce recombination. This structure is obtained when the conduction band minimum (CBM) of the ETL is higher than the CBM of the light absorber.^46,47^ Improvements in *V*_oc_ have also been achieved by modifying the ETL; for instance, pre-treating the SnO_2_ layer with SnCl_2_ for cells with Ag_2_BiI_5_ as the absorber enabled achieving a high *V*_oc_ of 0.81 V.^19^ The formation of an amorphous SnO_*x*_ layer has been proven to reduce charge recombination at the interface.^47^ Doping AgBiI_4_ with lithium bis(trifluoromethanesulfonyl)imide (Li-TFSI) also improved the *V*_oc_ for planar heterojunctions, significantly enhancing the FF; in this case, TFSI^−^ anions are responsible for improving the morphology, assisting in the film growth.^31^ Devices with different configurations have demonstrated enhanced *V*_oc_ values. For example, when a compact layer of TiO_2_ and carbon electrodes were employed in Ag_2_BiI_5_ devices treated with air blowing, the open-circuit voltage reached 0.77 V.^39^ Furthermore, in the case of planar inverted Ag_3_BiI_6_ devices using NiO_*x*_ and PCBM/C_60_, the open-circuit voltage achieved values of 0.82 V and 0.77 V, respectively.^48^

Comparatively, the *J*_sc_ values are subject to change considerably more than the *V*_oc_ and FF values. Therefore, improving *J*_sc_ is pivotal to enhancing the conversion efficiency of SBI solar cells. Three major strategies have been advocated for this purpose. The first strategy involves enhancing *J*_sc_ by employing mesoporous structures instead of planar heterojunctions, a method we have previously demonstrated.^49^ Second, replacing some of the materials in the SBI composition, as referred to in the “Others” category in Fig. 3, has proven to be beneficial. The highest efficiency among SBI-based solar cells has been achieved with the compound Ag_3_BiI_5.92_S_0.04_, obtained from a mixture of Ag_3_BiI_6_ and 4% bismuth(iii) tris(4-methylbenzodithioate) (C_24_H_21_BiS_6_, referred to as Bi(S_2_CAr)_3_ by the authors). Importantly, this sulfur-doped composition also exhibits higher *V*_oc_ and FF values compared to other compositions (Ag_3_BiI_6_, Ag_2_BiI_5_, AgBiI_4_, and AgBi_2_I_7_).^20^

Incorporating elements other than sulfur in the synthesis of SBI also demonstrated an improvement in solar cell efficiency. Specifically, the partial replacement with copper cations has been shown to increase the *J*_sc_ of Ag_2_BiI_5_ from 6.04 mA cm^−2^ to 7.13 mA cm^−2^. This enhancement is achieved by replacing a portion of AgI with copper iodide (CuI) as the starting material, resulting in mixed Ag_2−*x*_Cu_*x*_BiI_5_ (*x* = from 0.03 to 0.2), which leads to an increased absorption coefficient.^25^ The use of antimony has also been reported to enhance the morphology of AgBi_2_I_7_ and increase the conversion efficiency from 0.3% to 1.76%.^26^ Previous studies using halides other than iodine are relatively uncommon. However, it was observed that incorporation of Br into AgBiI_4_ led to the formation of AgBi(I_1−*x*_Br_*x*_)_4_, which improved the morphology and FF.^50^ Finally, employing bulk heterostructures has proven beneficial: the Ag_3_Bi_2_I_9_/CsBiI_9_ bulk heterojunction exhibits superior performance compared to standalone SBI device, improving both *V*_oc_ and *J*_sc_ and achieving a conversion efficiency of 3.59%.^51^

Up to this point, photoconversion layers composed of Ag–Bi–I even with appropriate material ratios and morphology have yet to match the high conversion efficiencies observed in organic–inorganic lead halide perovskite solar cells. The guidelines for achieving high efficiency in SBI solar cells remain largely unknown, suggesting that other crucial factors may be influencing the performance. This paper aims to elucidate the issues encountered with SBI films and discuss how each issue individually impacts the overall performance.

## Addressing issues

Since the state of the art has been outlined, this discussion focuses on the issues potentially impacting SBI solar cells. The sections are divided based on common issues identified by previous researchers, aiming to understand the underlying problems in applying SBI to solar cells.

### Crystal structure and composition

SBI crystals have been reported to exhibit two distinct crystal systems depending on their composition. For Ag-rich materials such as Ag_3_BiI_6_ or Ag_2_BiI_5_, a trigonal (rhombohedral) structure (*R*3̄*m*) is preferred, while a cubic structure (*Fd*3̄*m*) is observed in Bi-rich materials like AgBi_2_I_7_ or AgBiI_4_. There is disagreement regarding the structure of Ag_2_BiI_7_, with initial studies suggesting that it forms a cubic structure akin to the ThZr_2_H_7_ type.^16^ However, Mitzi and Yan group showed that this structure is unstable due to the short Bi–I bonds and that Ag_2_BiI_7_ is likely to take the Ag-deficient AgBiI_4_ structure (cubic structure, *Fd*3̄*m*).^52^ Intrinsic investigation of the relationship between the composition and crystal structure has been conducted using Ag_4−6*x*_Bi_2*x*_I_4_, and a rhombohedral to cubic transition was observed depending on the AgI : BiI_3_ ratio used.^53^ From the above, it is expected that the Ag–Bi–I crystalline material is a thermodynamically random solid solution of each ion and that the resulting crystalline system varies due to slight compositional deviations associated with temperature effects.^54,55^

**Fig. 4:**
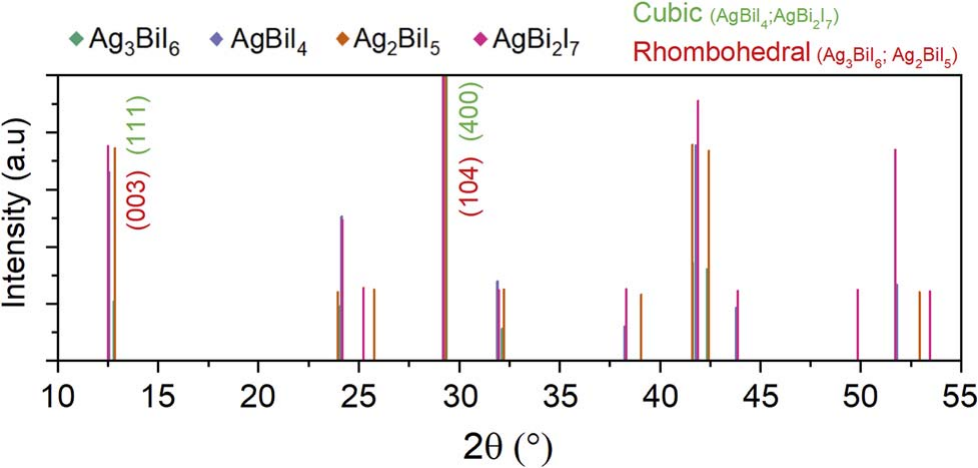
XRD patterns extracted from crystallographic libraries for SBI compounds. Ag_3_BiI_6_ (CCDC 1776740)^62^ (blue), AgBiI_4_ (CCDC 1671019)^62^ (violet), Ag_2_BiI_5_ (JCPDS card # 00-035-1025) (orange) and AgBi_2_I_7_ (PDF card # 00-034-1372) (pink).

The potential for the formation of multiple SBI phases, utilizing AgI and BiI_3_ as starting materials, warrants consideration. Mashadieva *et al.* have highlighted in their study on the AgI–BiI_3_ system that Ag_3_BiI_6_ and AgBiI_4_ might constitute a mixture of Ag_2_BiI_5_ and AgBi_2_I_7_.^55^ The occurrence of various SBI compounds in AgBiI_4_ films^43^ has been documented previously, and the formation of Ag_3_BiI_6_ and AgBiI_4_ is also anticipated based on calculations.^56,58^ It should be emphasized that X-ray diffraction (XRD) measurements might not discern SBI crystals of varying compositions when mixed, due to the similarity in SBI peaks (Fig. 4). Therefore, one issue is the potential formation of multiple SBI phases during synthesis of a specific SBI film. The small variation in bandgaps among SBI compounds may result from the coexistence of multiple Ag–Bi–I structures. Such heterogeneous systems in SBI may be equivalent to phase segregation observed in perovskites, which is detrimental to carrier transport and overall solar cell performance,^57^ but first it is necessary to confirm SBI purity upon synthesis.

On the other hand, based on the XRD results, it is observed that the SBI compound exhibits two main peaks, primarily located around 12° and 29° (Fig. 4). Depending on whether the structure is rhombohedral or cubic, these peaks are attributed to the (003) and (104) planes, or the (111) and (400) planes, respectively. Simulations predict that the peak at 29° is more intense than that at 12°.^56,58^ However, numerous studies have reported the opposite.^21,30,31,39^ It is noteworthy that higher *J*_sc_ values are often reported when the experimental XRD pattern aligns with the theoretical XRD pattern, specifically when the intensity of the 29° peak surpasses that of the 12° peak.^20,25,35,51^ We posit that enhancing crystallinity and the growth of SBI crystals may be a pivotal method to improve their properties. As it has already been observed in LHP,^59,60^ it could be possible that SBI crystals may exhibit different electrical properties depending on the plane, with the (104) plane potentially offering superior electrical properties compared to the (003) plane. Single crystals are essential for measuring the intrinsic properties of a single plane, and to date, this has been achieved solely through the vertical Bridgman method^61^ or the solvothermal method.^62^ Developing a technique to fabricate single crystals from solution with ease is imperative to further the research. Moreover, undertaking a study dedicated to SBI crystallization, particularly on how properties are influenced by the growth of specific planes or SBI purity, would be beneficial. As advocated, a thorough future analysis of SBI necessitates the incorporation of advanced analytical techniques that have yet to be applied. For instance, it has been established that Grazing Incidence Wide Angle X-ray Scattering (GIWAXS)^63,64^ is instrumental in evaluating the orientation of SBI crystals.^19^ Additionally, it is posited that Transmission Electron Microscopy (TEM)^65^ and Electron Backscatter Diffraction (EBSD)^66^ techniques will be beneficial in the future for accurately characterizing the mixed phases of SBI crystals. However, it is important to consider the potential damage caused by the electron beam.

### Ag^0^ and Bi^0^ impurities

Impurities in AgI and BiI_3_ can obstruct crystallization and lead to degradation in quality.^67^ The existence of metallic bismuth (Bi^0^) in SBI films has been confirmed.^20,36,37^ Notably, Bi^0^ is known to induce bismuth vacancies, adversely impacting the performance of SBI solar cells.^17^ Conversely, the origin of Bi^0^ remains a subject of debate. Given the weak nature of Bi–I bonds, Bi^0^ may form during the crystallization process of the SBI layer.^37^ Additionally, we identified ion migration as a potential cause for the production of Bi^0^.^36^ The possible presence of Bi^0^ in the contaminated reactants cannot be overlooked. The significance of starting materials' high purity for fabricating solar cells with elevated reproducibility and conversion efficiency has been substantiated for Pb^68,69^ and Sn^70,71^ based solar cells. Given the impact of Pb^0^ on the valence band edge, which induces trap states within the material,^72–74^ it is reasonable to surmise that the presence of Bi^0^ would similarly influence the valence band edge, markedly affecting the solar cell's properties.

Similarly, the formation of Ag^0^ poses a significant issue due to its role in charge recombination, negatively impacting the device performance. Ag^0^ can be formed from AgI, which may remain as an unreacted impurity in SBI. AgI, a material known for its photographic properties, is sensitive to visible light following the reversible reaction Ag^+^ + e_free_^−^ ↔ Ag^0^.^75^ Through the photographic mechanism, photoexcited free electrons in AgI could be trapped by interstitial Ag^+^ ions, leading to the formation of Ag. Although this reaction is reversible, both interstitial Ag^+^ ions and Ag^0^ can act as recombination centers, reducing the mobility of electrons and holes within the film. XRD analysis of SBI films has revealed trace amounts of AgI as an impurity, depending on the preparation method.^17–22,36,53,54,76–79^ This underscores the necessity of using high-purity AgI for preparing SBI films to enhance their efficacy as photovoltaic absorbers.

### Electron–phonon coupling

Electron–phonon coupling describes how lattice vibrations (phonons) interact with electrons in a solid material. This kind of interaction can explain different properties of semiconductors, but it is mainly important to understand their transport properties. In general, strong phonon scattering detrimentally affects charge-carrier mobility, weakens photoluminescence (PL) and increases recombination.

**Fig. 5:**
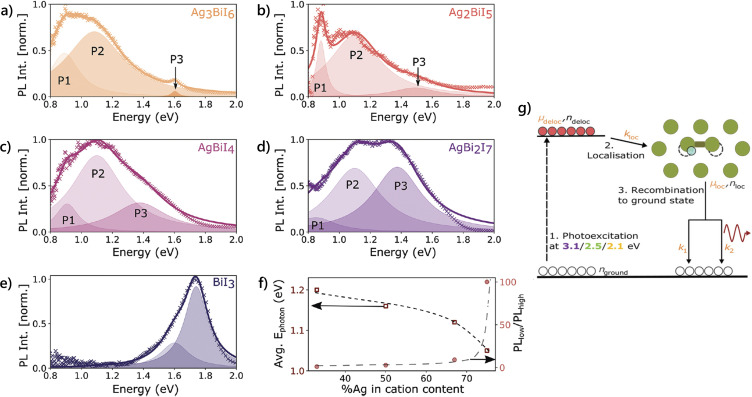
Photoluminescence (PL) spectra for SBI compounds (a) Ag_3_BiI_6_, (b) Ag_2_BiI_5_, (c) AgBiI_4,_ (d) AgBi_2_I_7_ and (e) BiI_3_ measured following 532 nm continuous wave excitation. The measured PL was detected using a cooled Si-CCD in the high-energy range (600–1100 nm) and a cooled InGaAs detector in the low-energy range (950–1600 nm). The PL was fit with a sum of several Lorentzian functions shown in the colored solid line with the individual Lorentzians plotted as shaded area curves. (f) Composition-dependent variation in PL_low_/PL_high_ (round markers, right axis) calculated as the ratio of the high-energy contribution (area under peak P_3_) and the low-energy contribution (sum of areas under peaks P_1_ and P_2_). Composition-dependent average photon energy *E*_photon_ (square markers, left axis) calculated as the intensity weighted average of the photon emission energy (=Σ*N*_*λ*_*E*_*λ*_/Σ*N*_*λ*_, where *N*_*λ*_ is the number of detected photons with energy lying within an interval of width Δ*E* centered at *E*_*λ*_, which corresponds to the PL intensity measured at *E*_*λ*_). Dashed lines are guides to the eye. Reproduced from ref. 86 with permission from Wiley-VCH GmbH, copyright 2024. (g) Schematic of the charge-carrier dynamic processes present across Cu_4*x*_(AgBi)_1−*x*_I_4_. *μ*_deloc_ and *μ*_loc_ correspond to the mobility of delocalized and localized charge carriers, respectively, while *k*_loc_ is the localization rate. Reproduced from ref. 81 with permission from Wiley-VCH GmbH, copyright 2021.

Bismuth-based materials have already been reported to have self-trapping and low carrier mobility due to the interaction of photoexcited carriers with the lattice.^80–86^ Their presence is related to a low electronic dimensionality of the material, thereby facilitating interactions between charge carriers and the lattice.^80,82^ In addition, the “soft” nature of silver–halide bonds and the facile structural distortion of bismuth induce the localization of charge carriers through the formation of small polarons.^56,81^ As a result, a strong electron–phonon interaction is anticipated, leading to the self-trapping of charge, weak PL, and low photovoltaic performance. Specifically, self-trapping or self-localization refers to a charge carrier that becomes trapped due to its interaction with the lattice, leading to the generation of a phonon (Fig. 5). This phenomenon depends on the chemical composition, dimensionality and capacity of structural distortion.^80^

In SBI compounds, such as Ag_3_BiI_6_, Ag_2_BiI_5_, AgBiI_4_ and AgBi_2_I_7_, strong electron–phonon interactions have also been observed.^83,86^ This phenomenon is linked to the presence of segregated Ag-rich and Bi-rich domains, which are responsible for charge carrier localization and exciton self-trapping in SBI.^86^ Moreover, Lal *et al.* observed a broad PL emission, which is associated with the strength of charge carrier localization (Fig. 5).^86^ Increasing the Ag ratio in SBI leads to weaker charge carrier localization and a decrease in PL emission, whereas Bi-rich compounds exhibit stronger charge carrier localization and higher PL emission.

On the other hand, ultrafast spectroscopic measurements revealed carrier-phonon dynamics, showcasing the rapid relaxation of charge carriers towards the band edge, particularly in AgI domains, with nonradiative relaxation occurring swiftly within a constant below 10 ps after photoexcitation, potentially triggering the formation of metallic silver over time from deeply trapped electrons with Ag^+^.^83^ Both mechanisms respond to electron–phonon dynamics that may affect *V*_oc_ in SBI solar cells through trap-assisted recombination.

These experiments align with earlier studies recognizing the limited carrier mobility in SBI films,^19,37,83,86^ as a result of electron–phonon coupling. Low charge mobility has been found to be associated with self-localization in SBI.^86^ Furthermore, this consistency is reflected in our observation of an enhanced *J*_sc_ in reported studies when employing a mesoporous structure compared to planar heterojunction devices.^17,20,22,25,32^ Similar to the dynamics observed in dye-sensitized solar cells, the mesoporous structure, with its augmented surface area, facilitates efficient charge collection and injection into the transport layers.^49^

As mobility is inversely proportional to the effective mass,^87,88^ replacing bismuth with cations to reduce the effective mass could potentially increase mobility. Likewise, incorporating different elements such as Cu^+^, Tl^3+^, or Sb^3+^ may enhance electronic connectivity, as silver and bismuth cations are commonly observed to exhibit low electronic dimensionality.^81,89^ Electronic dimensionality refers to the degree of connectivity between atomic orbitals that constitute the lower conduction band (LCB) and upper valence band (UVB).^90^ In SBI, the conduction band minimum (CBM) is derived from bismuth and iodine p orbitals, while the valence band maximum (VBM) is composed of silver d orbitals and iodine orbitals.^91^ This electronic configuration is linked to the edge-sharing octahedra crystal structure,^92^ which results in low connectivity on the conduction band and valence band orbitals, leading to SBI's low electronic dimensionality.

In actuality, it can be seen that the highest performance is exhibited by the SBI mixed structures, where a portion of iodine is substituted with sulfur (S), suggesting a potential association with the improved mobility. Moreover, incorporating ions or molecules capable of contributing to the valence and conduction bands has the potential to enhance the electrical connectivity,^90^ thereby potentially improving carrier transport. The standalone use of SBI materials may not be suitable for solar cell applications, and we suggest the incorporation of ions into SBI films while simultaneously reducing their effective mass as a promising approach for improvement.

### Recombination in SBI

Self-trap recombination, as previously discussed, is evident from the electron–phonon dynamics within SBI. Recombination rates for Ag_3_BiI_6_ and AgBiI_4_ have been recorded as 4.32 × 10^8^ s^−1^ and 2 × 10^8^ s^−1^, respectively.^83^ The comparatively slower recombination rate for Ag_3_BiI_6_ relative to that of AgBiI_4_ could account for its superior performance,^51,83^ aligning with the distribution of characteristics of solar cell devices illustrated in Fig. 3. Self-trapping is further induced by point defects within the SBI crystal, including vacancies of silver, bismuth, and iodine. Additionally, BiAg antisites have been identified as trapping sites.^81^ Moreover, vacancies in the SBI structure can further distort the crystal lattice, enhancing electron–phonon coupling. A higher concentration of vacancies has been observed in Bi-rich compounds, contributing to vacancy-mediated lattice softening. This, in turn, leads to stronger charge carrier localization or self-trapped recombination, ultimately reducing carrier mobility.^86^

It has been generally observed that compositions rich in Bi are prone to a higher incidence of vacancy sites, which correlates with lower *V*_oc_ values.^93^ The *V*_oc_ values of SBI solar cells demonstrate a trend wherein configurations rich in Ag exhibit approximately 0.1 V higher than those rich in Bi. This tendency is likely due to the formation of vacancy sites originating from the generation of metallic bismuth (Bi^0^).

It is observed that the current SBI solar cells exhibit a higher *V*_oc_ loss compared to lead-based perovskite solar cells.^93,94^ The formation of small polarons is suggested as a self-trapping phenomenon within the SBI, potentially contributing to performance degradation. Additionally, the ratio of AgI to BiI_3_ and the generation of point defects during the crystallization process further complicate the issue. A thorough understanding of these aspects could contribute to mitigating voltage drop in SBI compounds.

In our previous research, we explored the behavior of SBI under low light intensities, uncovering the occurrence of bimolecular recombination (between conduction band electrons and valence band holes) in Ag_2_BiI_5_.^19^ Considering previous research, this observed phenomenon may be related to self-trapping in SBI. Although some studies advocate for an optimal thickness of 300 nm for Ag_3_BiI_6_ and AgBiI_4_,^58^ our findings suggest that increasing the thickness of the absorber leads to increased recombination, resulting in diminished properties.^19^ This observation is consistent with the reported detrimental effects associated with reduced mobility as the thickness of the absorber layer increases in LHP.^95^

Bimolecular recombination is more likely to occur in absorbers characterized by low mobility^96^ and is less affected by material processes. Buizza *et al.* have documented the emergence of small polaron formation in the copper–silver–bismuth system, which promotes self-localization in Cu_4*x*_(AgBi)_1−*x*_I_4_.^81^ However, they encountered difficulties in identifying a model that corresponds to both experimental outcomes and theoretical expectations. As a result, they propose the occurrence of Langevin-type recombination on the film. Similar behaviour was previously observed in organic solar cells, where discrepancies between recombination constants obtained *via* simulation and experimental methods were noted.^97^ To address this difference, a carrier charge concentration gradient may be suggested to explain the disparity between recombination constants.^97^ However, further research on bismuth-based materials remains limited. For SBI compounds, with small-polaron formation, recombination might occur on a similar basis as documented for the copper–silver–bismuth system. From our observations^19^ and the established understanding that bimolecular recombination limits the short-circuit current,^63^ the noted reduction in *J*_sc_ with an increasing Bi ratio in SBI solar cells may indicate a heightened incidence of recombination in Bi-rich compounds. Specifically, recombination occurs by self-localization of charges due to electron–phonon coupling. The crystal structure and capacity for distortion play a key role in determining the localization strength. Low mobility, and consequently low PCE, in Bi-rich compounds is associated with a higher vacancy fraction, which increases the susceptibility of SBI to distortions and, in turn, enhances electron–phonon interactions.^86^

## Perspectives

SBI solar cells represent a promising lead-free alternative to lead-based perovskite solar cells, but they require further enhancements to emerge as a viable option. Various studies have shed light on methods to improve the conversion efficiency of SBI solar cells. We assert that adhering to the following strategies could substantially elevate their performance. Primarily, it is crucial to understand the crystallization process of SBI and its direct impact on physical properties. This understanding could improve the reproducibility of the fabrication process and pinpoint areas for improvement. Secondly, given the role of phonon dynamics in potentially limiting the open-circuit voltage, substituting appropriate cations within the SBI framework could diminish recombination rates and enhance carrier extraction efficiency. Additionally, selecting a suitable charge transport layer that aligns with the energy levels is imperative. Herein, we outline our perspective on the future direction for SBI solar cell enhancement.

### Crystallization process and crystal analysis

The comprehension of the crystallization process in the fabrication of SBI crystal layers is paramount. During the solution process, it is essential to investigate crystallization while heating, as this is key to producing larger crystals and aids in understanding solvent interactions. Exploring additives that could facilitate crystallization represents a promising avenue for further research. Additives such as Lewis bases^98^ can control crystallization in order to obtain improved SBI films. Aside from solution-based methods, synthesis of SBI can be obtained *via* a solid-state reaction, grinding and calcination,^99^ which can reduce impurities due to low solubility.

Regulating the growth of specific SBI crystal planes is another critical factor. Selectively generating (003) and (104) planes and evaluating their influence on the charge separation process are fundamental. Moreover, the development of techniques and the enhancement of understanding for the accurate assessment of SBI crystals are essential. While multiple SBI compounds may exist, the precise identification of these compounds using XRD is challenging due to resolution limitations. Furthermore, techniques such as Electron Backscatter Diffraction (EBSD)^100^ and Transmission Electron Microscopy (TEM) could be appropriate for directly determining the SBI crystal system. However, these techniques involve high-energy measurements, which pose a risk of damaging SBI crystals, necessitating careful handling during analysis.^65^

On the other hand, X-ray Photoelectron Spectroscopy (XPS) is a valuable method for determining the Ag : Bi : I ratio. This technique not only enables the measurement of elemental composition but also simultaneously identifies the valence states of each element. It is effective in detecting metallic states such as Ag^0^ and Bi^0^. Additionally, a notable advantage of this method is its capability for mapping analysis. Furthermore, Kelvin Probe Force Microscopy (KFM) is a promising technique for verifying the presence of materials with different compositions,^101^ as it facilitates the visualization of potential differences. In addition, bulk techniques such as inductively coupled plasma mass spectrometry (ICP-MS) and inductively coupled plasma optical emission spectrometry (ICP-OES), in combination with spatially resolved techniques such as laser ablation ICP-MS (LA-ICP-MS), are considered promising tools for measuring the Ag : Bi : I ratio.

### Improving mobility and decreasing recombination

The limited charge mobility in SBI is ascribed to pronounced electron–phonon coupling. Improvements in charge mobility are achieved by enhancing the grain size through solution engineering; however, even with low mobility, charge collection efficiency can be improved through strategic decisions regarding the device structure, such as employing mesoporous structures. The introduction of mesoporous materials enhances carrier collection and provides an opportunity to explore promising materials, such as mesoporous SnO_2_ ^102^ and nanotubes,^103^ to enhance carrier transport. Moreover, strategies for synthesizing uniformly dispersed nanocrystals, as exemplified by AgBiS_2_, can enhance electrical dimensionality, ultimately leading to higher charge mobility within the film.^104^

Furthermore, the presence of AgI can lead to the formation of Ag^0^, which may act as a recombination center, necessitating a reduction in impurity content within the film. It has been observed that solution processing induces the formation of AgI impurities due to the limited solubility of precursor materials. In this regard, a mixed DMSO : DMF solvent system has been shown to enhance the solubility of AgI compared to DMF alone. Moreover, the use of additives or alternative solvents, such as the incorporation of iodine, has been reported to enhance AgI solubility,^105,106^ thereby facilitating improved crystallization. On the other hand, solid-state reaction methods have exhibited a significant reduction in AgI impurities, suggesting that this approach could be a viable alternative for fabricating SBI films with higher purity.

### Decreasing electron–phonon coupling behaviour

“Pure” SBI, composed solely of Ag, Bi, and I, might not be a practical option due to the possible impediment of charge transfer by polaron formation, leading to a reduction in conversion efficiency. Reduction in effective mass could be realized through the cation exchange process, in which a portion of Ag^+^ and/or Bi^3+^ ions are replaced by ions such as Cu^+^, Rb^+^, and/or Sb^3+^. The use of a mixed structure with SBI serving as the base framework offers diverse pathways for carrier movement, thereby enhancing charge transfer efficiency. Moreover, since vacancies may be responsible for stronger polaronic effects that enhance self-localization and decrease mobility in SBI,^86^ it is primordial to obtain SBI films without impurities that could lead to the formation of vacancies.

Recently, it has been shown that applying pressure to Cs_2_AgBiCl_6_ diminishes electron–phonon coupling, maintaining the crystal structure and enhancing the PL lifetime.^107^ Investigating whether SBI crystals exhibit similar behavior could offer a novel avenue to boost the performance of photovoltaic systems.

### Scaling up SBI

The research on SBI-based solar cells remains in its early stages; however, it is crucial to consider the feasibility of scaling up for future large-area applications. One of the primary challenges in upscaling is the presence of AgI impurities, which become more likely as the active area expands, ultimately leading to a decline in efficiency. Therefore, controlling impurity formation during synthesis and optimizing film uniformity are essential steps to ensure stable performance in larger area devices.

Another critical factor in scaling up is the selection of an appropriate fabrication method. While conventional solution processes such as spin coating are effective for small scale fabrication, they may not be suitable for large-area production.^108^ Alternative deposition techniques, such as inkjet printing, could serve as a viable approach for the coating process in SBI thin films. Therefore, careful consideration should be given to the significant impact of the low solubility of AgI. The strategic development of additives that improve the solubility of AgI may be the key to achieving large-area fabrication of solar cells.

## Conclusions and outlook

SBI crystals, being solely composed of inorganic materials, exhibit relative resistance to moisture and oxygen, suggesting their potential for excellent durability. This characteristic is particularly relevant for flexible device applications, where plastic film substrates are permeable to oxygen and moisture. Moreover, compared to other lead-free materials, SBI still exhibits lower efficiency compared to other lead-free materials. Currently, silver bismuth sulfide (AgBiS_2_) solar cells achieve efficiencies of around 9%,^104^ while Cs-based perovskites have reached 11%.^109^

Given its bandgap and documented success in indoor applications,^19,110,111^ SBI's utility could be optimally exploited in this domain, contrasting with terrestrial applications where perovskites are viewed as formidable contenders. To harness this potential, selecting a suitable HTL and ETL tailored for indoor conditions is imperative.

The most pressing issue is the low conversion efficiency; addressing this challenge requires a more comprehensive understanding of the fundamental properties of SBI, including its formation process and its effect on efficiency. It is essential to investigate the presence of multiple SBI compounds in the film and determine whether the optoelectronic properties vary depending on the growth orientation of SBI. These fundamental insights will provide the foundation for developing effective strategies to enhance performance.

For now, the incorporation of mixed structures has shown potential for improving efficiency by enhancing charge carrier mobility. Furthermore, decreasing impurities on SBI films and the use of mixed structures can mitigate the effects of electron–phonon coupling.^92^ Through continuous and innovative research efforts in compositional and interfacial engineering to improve carrier mobility, there is promising potential to overcome and address this significant challenge.

## Data availability

Data for this article, including Table S1 of SBI efficiencies and parameters through the years are available at ESI at https://doi.org/10.1039/D4SC07955H.†

## Author contributions

N. B. C. G. and S. N. conceived the idea. N. B. C. G. conducted the literature review, analysed the published results, and wrote the manuscript. T. M. and S. N. provided key advice and supervised the preparation of the text. All authors discussed and commented on the manuscript.

## Conflicts of interest

There are no conflicts to declare.

## Supplementary Material

SC-016-D4SC07955H-s001
