# Supplementary material for: Advancements and prospects for eco-friendly, high-performance silver bismuth halide solar cells
Source: Chem Sci. 2025 Mar 6;16(14):5807–18. doi: 10.1039/d4sc07955h (PMC11912501; doi:10.1039/d4sc07955h)
Supplement: SC-016-D4SC07955H-s001 [file SC-016-D4SC07955H-s001.pdf]

Supplementary Information (SI) for Chemical Science.
This journal is © The Royal Society of Chemistry 2025

Supporting information

Advancements and Prospects for eco-friendly, high-performance silver bismuth halide solar cells

Natalia Belen Correa Guerrero [a,b], M. Dolores Perez[b]*, Naoyuki Shibayama[a]*, Tsutomu Miyasaka[a,c]*

[a] Toin University of Yokohama, 1614 Kurogane-cho, Aoba, Yokohama, Kanagawa, Japan

[b] Instituto de Nanociencia y Nanotecnología, CNEA-CONICET, Centro Atómico Constituyentes, Avda. Gral. Paz 1499, San Martín 1650, Buenos Aires, Argentina

[c] Research Center for Advanced Science and Technology (RCAST), The University of Tokyo, 4-6-1 Komaba, Meguro-ku, Tokyo, Japan

AUTHOR INFORMATION

*Corresponding Author

M. Dolores Perez: mdperez@unsam.edu.ar

Naoyuki Shibayama: shibayama@toin.ac.jp

Tsutomu Miyasaka : miyasaka@toin.ac.jp

Table S1. Reported Silver Bismuth Iodide (ABI) solar cells through the previous years.

Year	ABI	Configuration	Jsc mA/cm²	V_{oc} V	FF	PCE %	Method	Solvent Antisolvent (AS)	Ref.
2016	$AgBi_2I_7$	FTO/TiO_2*/$AgBi_2I_7$/ P3HT/Au	3.3	0.56	0.674	1.22	spin coating	n-butylamine	1
2016	$AgBi_2I_7$	$FTO/TiO_2/AgBi_2I_7$/ P3HT/Au	4.83	0.62	0.70	2.12	spin coating	DMSO	2
2017	Ag_3BiI_6	$FTO/TiO_2/Ag_3BiI_6$/ PTAA/Au	10.7	0.63	0.64	4.3	spin coating AS: toluene	DMSO:DMF	3
2017	Ag_2BiI_5	$FTO/TiO_2/Ag_2BiI_5$/ P3HT/Au	5.8	0.49	0.63	2.1	spin coating	butylamine	4
2017	$AgBi_2I_7$	$FTO/TiO_2/AgBi_2I_7$/ P3HT/Au	1.6	0.46	0.58	0.4	spin coating	butylamine	4
2018	$AgBiI_4$	FTO/TiO_2/AgBiI4/ PTAA/Au	3.7	0.63	0.514	1.2	spin coating	DMSO:DMF (1:1) AS: chlorobenze	5
2018	$AgBiI_4$	$FTO/TiO_2/AgBiI_4$/ PTAA/Au	5.24	0.67	0.621	2.2	dynamic hot casting	DMSO:DMF (1:1) AS: chlorobenze	5
2018	Ag_2BiI_5	$FTO/TiO_2/Ag_2BiI_5$ /PTAA/Au	4.42	0.63	0.576	1.6	spin coating	DMSO:DMF (1:1) AS: chlorobenze	5
2018	Ag_2BiI_5	$FTO/TiO_2/Ag_2BiI_5$/ PTAA/Au	6.04	0.69	0.624	2.6	dynamic hot casting	DMSO:DMF (1:1) AS: chlorobenze	5
2018	Ag_3BiI_6	$FTO/TiO_2/Ag_3BiI_6$/ PTAA/Au	11.2	0.607	0.646	4.33	Ar air blowing spin coating	DMSO:DMF:HI (3:1)	6
2018	$AgBiI_4$	$FTO/TiO_2/AgBiI_4$/ PTAA/Au	8.07	0.562	0.523	2.36	Ar air blowing spin coating	DMSO:DMF:HI (3:1)	6
2018	Ag_2BiI_5	$FTO/TiO_2/Ag_2BiI_5$ /PTAA/Au	8.9	0.522	0.681	3.16	Ar air blowing spin coating	DMSO:DMF:HI (3:1)	6
2018	$AgBi_2I_7$	$FTO/TiO_2/AgBi_2I_7$/ PTAA/Au	5.8	0.599	0.597	2.09	Ar air blowing spin coating	DMSO:DMF:HI (3:1)	6
2018	Other	$FTO/TiO_2/Ag_3BiI_{5.92}$ $S_{0.04}$/PTAA/Au	14.7	0.573	0.659	5.56	Ar air blowing spin coating	DMSO:DMF:HI (3:1)	6
2018	Other	$FTO/TiO_2/AgBiI_{3.92}$ $S_{0.04}$/PTAA/Au	9.46	0.527	0.55	2.75	Ar air blowing spin coating	DMSO:DMF:HI (3:1)	6
2018	Other	$FTO/TiO_2/Ag_2BiI_{4.92}$ $S_{0.04}$/PTAA/Au	13.1	0.479	0.624	3.91	Ar air blowing spin coating	DMSO:DMF:HI (3:1)	6
2018	Other	$FTO/TiO_2/AgBi_2I_{6.92}$ $S_{0.04}$/PTAA/Au	7.68	0.569	0.566	2.48	Ar air blowing spin coating	DMSO:DMF:HI (3:1)	6
2019	$AgBiI_4$	$ITO/SnO_2/AgBiI_4$/ PTAA/Au	4.26	0.66	0.591	1.67	spin coating	DMSO:DMF (4:1)	7
2019	$AgBiI_4$	$ITO/SnO_2/AgBiI_4$:Li -TFSI2%/PTAA/Au	5.07	0.83	0.665	2.8	spin coating	DMSO:DMF (4:1)	7
2020	Ag_2BiI_5	$FTO/TiO_2/Ag_2BiI_5$/ PTB7/MoO_x/Ag	3.77	0.49	0.59	1.26	spin coating	DMSO:HI (5%vol)	8
2020	Ag_2BiI_5	FTO/TiO2/Ag_2BiI_5/ PTB7/MoOx/Ag	2.64	0.59	0.37	0.78	spin coating	DMSO	8
2020	Other	FTO/TiO_2/ Ag-(Bi-Sb)-I (3:1:1:9)/PTB7/ MoO_x/Ag	6.05	0.52	0.58	1.82	spin coating	DMSO	8
2020	$AgBi_2I_7$	$FTO/TiO_2/AgBi_2I_7$/ PTB7/MoO_x/Ag	0.91	0.67	0.51	0.31	spin coating	DMSO:DMF (1:1)	9

2020	Other	FTO/TiO_2/ $AgBi_{1.5}Sb_{0.5}I_7$/PTB7/ MoO_x/Ag	2.27	0.57	0.43	0.56	spin coating	DMSO:DMF (1:1)	9
2020	Other	FTO/TiO_2/ $AgBiSbI_7$/PTB7/ MoO_x/Ag	2.11	0.55	0.5	0.58	spin coating	DMSO:DMF (1:1)	9
2020	Other	FTO/TiO_2/ $AgBi_{0.5}Sb_{1.5}I_7$/PTB7/ MoO_x/Ag	5.66	0.53	0.59	1.76	spin coating	DMSO:DMF (1:1)	9
2020	Ag_3BiI_6	FTO/TiO_2/Ag_3BiI_6/ NiO/Au	0.36	0.65	0.33	0.08	DC Sputtering	-	10
2020	Ag_3BiI_6	ITO/NiO_x/Ag_3BiI_6/ PCBM/C60/Au	1.08	0.77	0.37	0.31	spin coating	n-butylamine	11
2020	Ag_3BiI_6	ITO/NiO_x/Ag_3BiI_6/ PCBM/C60/Au	1.73	0.82	0.76	1.08	dynamic spin coating	n-butylamine	11
2020	Other	FTO/TiO_2/$Ag_3Bi_2I_9$/ PDBD-T/Au	4.9	0.7	0.578	1.97	spin coating	DMSO	12
2020	Other	FTO/TiO_2/ 0.5$Cs_3Bi_2I_9$- $Ag_3Bi_2I_9$/ PDBD-T/Au	7.65	0.78	0.601	3.59	spin coating	DMSO	12
2021	Ag_2BiI_5	FTO/c-TiO_2/ Ag_2BiI_5/Carbon	1.67	0.595	0.42	0.42	air blowing	DMSO:DMF (1:4)	13
2021	Ag_2BiI_5	FTO/c-TiO_2/ Ag_2BiI_5/Carbon	1.96	0.77	0.41	0.71	air blowing	DMSO:DMF (1:4)	13
2021	Ag_3BiI_6	FTO/TiO_2/Ag_3BiI_6/ P3HT/Au	5.12	0.58	0.61	1.8	spin coating	DMSO AS: chlorobenzene	14
2021	Ag_2BiI_5	FTO/TiO_2/Ag_2BiI_5/ P3HT/Au	5.65	0.54	0.67	2.04	spin coating	DMSO AS: chlorobenzene	14
2021	$AgBi_2I_7$	FTO/TiO_2/$AgBi_2I_7$/ P3HT/Au	5.05	0.56	0.74	2.1	spin coating	DMSO AS: chlorobenzene	14
2021	Ag_2BiI_5	FTO/TiO_2/Ag_2BiI_5/ Spiro-OMeTAD/Au	1.51	0.4	0.415	0.25	spin coating	DMF	15
2021	Ag_2BiI_5	FTO/TiO_2/Ag_2BiI_5/ Spiro-OMeTAD/Au	2.25	0.48	0.498	0.54	spin coating	DMSO:DMF (1:3)	15
2021	Ag_2BiI_5	FTO/TiO_2/Ag_2BiI_5/ Spiro-OMeTAD/Au	2.31	0.49	0.545	0.62	spin coating	DMSO:DMF (1:1)	15
2021	Ag_2BiI_5	FTO/TiO_2/Ag_2BiI_5/ Spiro-OMeTAD/Au	2.2	0.47	0.501	0.52	spin coating	DMSO:DMF (3:1)	15
2021	Ag_2BiI_5	FTO/TiO_2/Ag_2BiI_5/ Spiro-OMeTAD/Au	2.13	0.46	0.505	0.49	spin coating	DMSO	15
2021	Ag_2BiI_5	FTO/TiO_2/Ag_2BiI_5/ PTAA/Ag	6.04	0.602	0.561	2.04	spin coating	DMSO:DMF (3:2) AS: ethyl ether	16
2021	Other	FTO/TiO_2/ $Ag_{1.95}Cu_{0.05}BiI_5$/ PTAA/Ag	7.13	0.619	0.573	2.53	spin coating	DMSO:DMF (3:2) AS: ethyl ether	16
2021	Other	FTO/c-TiO_2/ $Ag_2Bi_3I_{11}$/P3HT/Au	2.65	0.34	0.49	0.44	spin coating	DMSO:DMF:HI	17
2021	Other	FTO/c-TiO_2/ $Ag_2Bi_3I_{11}$/Blue dye/P3HT/Au	4.98	0.52	0.58	1.5	spin coating	DMSO:DMF:HI	17
2022	Ag_3BiI_6	FTO/TiO_2/Ag_3BiI_6 /PTAA/Ag	5.34	0.71	0.623	2.36	spin coating	DMSO AS: chlorobenzene	18
2022	$AgBiI_4$	ITO/SnO_2/$AgBiI_4$/ PTAA/Au	2.77	0.47	0.5	0.64	spin coating	DMSO:DMF (1:1)	19
2022	$AgBiI_4$	ITO/SnO_2/$AgBiI_4$/ PTAA/Au	3.44	0.53	0.52	0.94	spin coating	DMSO:DMF (1:1) AS: chlorobenzene	19

2022	$AgBiI_4$	ITO/SnO_2/$AgBiI_4$/ PTAA/Au	3.58	0.53	0.58	1.11	spin coating	DMSO:DMF (1:1) AS: toluene	19
2022	$AgBiI_4$	ITO/SnO_2/$AgBiI_4$/ PTAA(Li-TFSI)/Au	3.89	0.58	0.56	1.26	spin coating	DMSO:DMF (1:1) AS: IPA	19
2023	Ag_2BiI_5	ITO/SnO_2/Ag_2BiI_5/ PTAA/Au	4.47	0.81	0.55	2.01	spin coating	DMSO:DMF (1:3)	20
2023	Ag_3BiI_6	ITO/TiO_2/Ag_3BiI_6/ PTAA(Li-TFSI)/Ag	6.44	0.7	0.617	2.77	doctor blade	DMSO	21
2023	$AgBi_2I_7$	FTO/TiO_2/$AgBi_2I_7$/ Spiro-OMeTAD/Au	3.07	0.65	0.48	0.96	spin coating	DMF:MeOH (1:1)	22
2023	$AgBi_2I_7$	FTO/TiO_2/$AgBi_2I_7$/ PTB7/Au	2.41	0.71	0.551	0.94	spin coating	n-butylamine	23
2024	$AgBiI_4$	FTO/TiO_2/$AgBiI_4$/ P3HT/Carbon	4.41	0.67	0.520	1.04	spin coating	DMSO:DMF (4:1)	24
2024	Ag_2BiI_5	FTO/TiO_2/Ag_2BiI_5/ Carbon	3.63	0.63	0.407	0.93	melt-solidification	DMSO:DMF (4:1)	25
2024	Ag_2BiI_5	FTO/TiO_2/Ag_2BiI_5/ Carbon	3.99	0.64	0.482	1.22	melt-solidification	DMSO:DMF (3:1)	26
2024	Ag_2BiI_5	FTO/TiO_2/Ag_2BiI_5/ Spiro-OMeTAD/Au	3.71	0.63	0.564	1.32	spin coating	DMSO	27
2024	$AgBiI_4$	FTO/TiO_2/$AgBiI_4$/ PEDOT:PSS/Ag	2.82	0.44	0.44	0.84	spin coating	DMSO:DMF (1:3) AS: toluene	28
2024	$AgBiI_4$	ITO/SnO_2/ $AgBiI_4$/ PTAA/Ag	2.99	0.78	0.577	1.87	spin coating	DMSO:DMF (1:1) AS: IPA	29

*: TiO_2 stands by c-TiO_2/m-TiO_2 if is not clarified.

References

1 Y. Kim, Z. Yang, A. Jain, O. Voznyy, G.-H. Kim, M. Liu, L. N. Quan, F. P. García de Arquer, R. Comin, J. Z. Fan and E. H. Sargent, *Angew. Chem. Int. Ed.*, 2016, **55,** 9586–9590.

2 A. Kulkarni, A. K. Jena, M. Ikegami and T. Miyasaka, *Chem. Comm.*, 2019, **55**, 4031–4034.

3 I. Turkevych, S. Kazaoui, E. Ito, T. Urano, K. Yamada, H. Tomiyasu, H. Yamagishi, M. Kondo and S. Aramaki, *ChemSusChem*, 2017, **10**, 3754–3759.

4 H. Zhu, M. Pan, M. B. Johansson and E. M. J. Johansson, *ChemSusChem*, 2017, **10**, 2592–2596.

5 B. Ghosh, B. Wu, X. Guo, P. C. Harikesh, R. A. John, T. Baikie, Arramel, A. T. S. Wee, C. Guet, T. C. Sum, S. Mhaisalkar and N. Mathews, *Adv. Energy Mater.*, 2018, **8**, 1802051.

6 N. Pai, J. Lu, T. R. Gengenbach, A. Seeber, A. S. R. Chesman, L. Jiang, D. C. Senevirathna, P. C. Andrews, U. Bach, Y. B. Cheng and A. N. Simonov, *Adv. Energy Mater.*, 2018, **9**, 201803396.

7 Q. Zhang, C. Wu, X. Qi, F. Lv, Z. Zhang, Y. Liu, S. Wang, B. Qu, Z. Chen and L. Xiao, *ACS Appl. Energy Mater.*, 2019, **2**, 3651–3656.

8 F. Iyoda, R. Nishikubo, A. Wakamiya and A. Saeki, *ACS Appl. Energy Mater.*, 2020, **3**, 8224–8232.

9 H. Zhu, A. Erbing, H. Wu, G. J. Man, S. Mukherjee, C. Kamal, M. B. Johansson, H. Rensmo, M. Odelius and E. M. J. Johansson, *ACS Appl. Energy Mater.*, 2020, **3**, 7372–7382.

10 A. Crovetto, A. Hajijafarassar, O. Hansen, B. Seger, I. Chorkendorff and P. C. K. Vesborg, *Chem. Mater.*, 2020, **32**, 3385–3395.

11 Y. Seo, S. R. Ha, S. Yoon, S. M. Jeong, H. Choi and D. W. Kang, *J. Power Sources*, 2020, **453**, 227903.

12 W. Hu, X. He, Z. Fang, W. Lian, Y. Shang, X. Li, W. Zhou, M. Zhang, T. Chen, Y. Lu, L. Zhang, L. Ding and S. Yang, *Nano Energy*, 2020, **68**, 104362.

13 F. He, Q. Wang, W. Zhu, D. Chen, J. Zhang, C. Zhang and Y. Hao, *Sol. St. Elect.*, 2021, **176**, 107950.

14 A. Kulkarni, F. Ünlü, N. Pant, J. Kaur, C. Bohr, A. K. Jena, S. Öz, M. Yanagida, Y. Shirai, M. Ikegami, K. Miyano, Y. Tachibana, S. Chakraborty, S. Mathur and T. Miyasaka, *Solar RRL*, 2021, **5**, 2100077.

15 M. S. Shadabroo, H. Abdizadeh, M. Shabani and M. R. Golobostanfard, *Inorg. Chem.*, 2021, **60**, 11110–11119.

16 J. W. Park, Y. Lim, K. Y. Doh, M. T. Jung, Y. I. Jeon, I. S. Yang, H. S. Choi, J. Kim, D. Lee and W. I. Lee, *Sustain. Energy Fuels*, 2021, **5**, 1439–1447.

17 S. Y. Lee, S. M. Yoo and H. J. Lee, *Chem. Lett*, 2021, **50**, 953–955.

18 M. C. Wu, Q. H. Wang, K. C. Hsiao, S. H. Chen, C. M. Ho, M. H. Jao, Y. H. Chang and W. F. Su, *Chem. Eng. J. Adv.*, 2022, **10**, 100275.

19 W. Zhai, L. Huang, X. Cui, G. Li, Z. Zhang, P. Chen, Y. Li, Y. Tang, L. Lin, Z. Yan and J. M. Liu, *J. Mater. Chem. C*, 2022, **10**, 5321–5327.

20 N. B. Correa Guerrero, Z. Guo, N. Shibayama, A. K. Jena and T. Miyasaka, *ACS Appl. Energy Mater.*, 2023, **6**, 10274–10284.

21 K. C. Hsiao, Y. F. Yu, C. M. Ho, M. H. Jao, Y. H. Chang, S. H. Chen, Y. H. Chang, W. F. Su, K. M. Lee and M. C. Wu, *Chem. Eng. J.*, 2023, **451**, 138807.

22 P. Kumar, K. Ahmad and S. M. Mobin, *Nanoscale Adv.*, 2023, **5**, 1624–1630.

23 T. G. Kwon, T. Kim and Y. Kim, *Electron. Mater. Lett.*, 2024, **20**, 165–172.

24 S. Subramani, A. K. Ramasamy, G. Rajamanickam, A. K. Chauhan and R. Perumalsamy, *Inorg. Chem. Commun.*, 2024, **159**, 111838.

25 R. Isaac Daniel, R. Govindaraj, P. Balaji Bhargav, A. K. Chauhan and P. Ramasamy, *J. Solid State Chem.*, 2024, **340**, 125026.

26 R. Isaac Daniel, R. Govindaraj, P. Balaji Bhargav, R. Anandha Krishnan, A. K. Chauhan and P. Ramasamy, *Mater. Sci. Eng. B*, 2025, **313**, 117968.

27 C. Zhao, Y. Yao, J. Li, S. M. Masawa, R. Shi, J. Xu and J. Yao, *Energy Technol.*, 2024, **12**, 2300916.

28 J. Lee, J. Park, B. C. Jeon, S. Lim, J. Y. Back, J. Kim and T. Moon, *ACS Sustain. Chem. Eng.*, 2024, **12**, 14396–14401.

29 W. Zhai, W. Zheng, P. Shi, L. Huang, X. Cui, P. Chen, Y. Tang, L. Lin, Z. Yan and J. M. Liu, *J. Phys. Chem. C*, 2024, **128**, 8154–8160.
